# Genome-Wide Analysis of Menin Binding Provides Insights into MEN1 Tumorigenesis

**DOI:** 10.1371/journal.pgen.0020051

**Published:** 2006-04-07

**Authors:** Peter C Scacheri, Sean Davis, Duncan T Odom, Gregory E Crawford, Stacie Perkins, Mohamad J Halawi, Sunita K Agarwal, Stephen J Marx, Allen M Spiegel, Paul S Meltzer, Francis S Collins

**Affiliations:** 1 Genome Technology Branch, National Human Genome Research Institute, National Institutes of Health, Bethesda, Maryland, United States of America; 2 Cancer Genetics Branch, National Human Genome Research Institute, National Institutes of Health, Bethesda, Maryland, United States of America; 3 Whitehead Institute, Cambridge, Massachusetts, United States of America; 4 National Institute of Diabetes and Digestive and Kidney Diseases, National Institutes of Health, Bethesda, Maryland, United States of America; 5 National Institute on Deafness and Other Communication Disorders, National Institutes of Health, Bethesda, Maryland, United States of America; Yale University, United States of America

## Abstract

Multiple endocrine neoplasia type I (MEN1) is a familial cancer syndrome characterized primarily by tumors of multiple endocrine glands. The gene for MEN1 encodes a ubiquitously expressed tumor suppressor protein called menin. Menin was recently shown to interact with several components of a trithorax family histone methyltransferase complex including ASH2, Rbbp5, WDR5, and the leukemia proto-oncoprotein MLL. To elucidate menin's role as a tumor suppressor and gain insights into the endocrine-specific tumor phenotype in MEN1, we mapped the genomic binding sites of menin, MLL1, and Rbbp5, to approximately 20,000 promoters in HeLa S3, HepG2, and pancreatic islet cells using the strategy of chromatin-immunoprecipitation coupled with microarray analysis. We found that menin, MLL1, and Rbbp5 localize to the promoters of thousands of human genes but do not always bind together. These data suggest that menin functions as a general regulator of transcription. We also found that factor occupancy generally correlates with high gene expression but that the loss of menin does not result in significant changes in most transcript levels. One exception is the developmentally programmed transcription factor, *HLXB9*, which is overexpressed in islets in the absence of menin. Our findings expand the realm of menin-targeted genes several hundred-fold beyond that previously described and provide potential insights to the endocrine tumor bias observed in MEN1 patients.

## Synopsis

In multiple endocrine neoplasia type I, absence of the nuclear factor menin gives rise to endocrine tumors by a mechanism that is poorly understood. Using state-of-the-art genome-wide chromatin-immunoprecipitation coupled with microarray analysis technology, this paper significantly enlarges our understanding of the role of menin by greatly extending the number of gene targets where menin binds. The authors show that while menin frequently colocalizes with a protein complex that modifies chromatin structure, menin can also bind to many other promoters by an alternative mechanism. They also present data that potentially implicate one of the menin target genes, *HLXB9,* in the endocrine specificity of tumorigenesis in multiple endocrine neoplasia, type 1. Further experiments to confirm the role of *HLXB9* in tumorigenesis are necessary and may help explain how the loss of a ubiquitously expressed tumor suppressor gene can give rise to tumors in specific tissues.

## Introduction

The hallmark of multiple endocrine neoplasia type I (MEN1), is the development of tumors in the parathyroid, anterior pituitary, and enteropancreatic endocrine cells. Other associations occasionally found in MEN1 patients include foregut carcinoids, facial angiofibromas, lipomas, collagenomas, meningiomas, and smooth muscle tumors. MEN1 patients typically inherit loss of function mutations in the *MEN1* gene, and tumors arise following loss of the remaining wild-type allele. Thus, the *MEN1* gene follows the classic “two-hit” tumor suppressor model first proposed by Knudson for retinoblastoma [1]. Somatic mutations in the *MEN1* gene are also frequently found in sporadic parathyroid adenomas, insulinomas, gastrinomas, and lung carcinoids [2–6], indicating that loss of the *MEN1* gene is a major contributor to the development and maintenance of many nonhereditary endocrine tumors. There is currently no prevention of or cure for MEN1 cancers, and cancer treatment options are generally limited to surgical interventions.

The protein product of *MEN1,* menin, is a ubiquitously expressed 67-kDa protein found predominantly in the nucleus [7]. Studies in *Men1* knockout mice support menin's role as a tumor suppressor [8,9]. Mice that are homozygous null for *Men1* show developmental defects and die at embryonic day 11.5 to 13.5. Heterozygous *Men1* mice eventually develop multiple endocrine tumors that arise following somatic loss of the wild-type *Men1* allele, and the spectrum of tumors observed in *Men1^+/^* ^−^ mice is remarkably similar to that in human MEN1 kindreds. We and others have also successfully developed conditional knockouts of the *Men1* gene, using the *Cre-lox* system [10–12]. When homozygous *Men1* “floxed” mice are bred to animals expressing Cre recombinase in the beta cells of the pancreatic islets, the resultant progeny develop multiple pancreatic insulinomas. These insulinomas arise long after homozygous inactivation of the *Men1* gene, suggesting that additional somatic events are required for frank tumor formation. *Men1* ^−/−^ insulinomas are capable of developing in the absence of chromosomal or microsatellite instability, suggesting that the additional somatic events required for tumor formation are subtle, occurring at either the nucleotide or epigenetic level [13].

Although inherited mutations in the *MEN1* gene predispose individuals to several types of tumors, there is a particularly striking predisposition to tumors in endocrine tissues. The development of tumors in this endocrine-specific pattern is quite puzzling as menin appears to be expressed in all tissues. In a previous study, we set out to gain insights to this issue of tissue-specificity in MEN1 by breeding floxed *Men1* mice to transgenic mice expressing *Cre* recombinase from the albumin promoter, which is predominantly expressed in liver hepatocytes [14]. This strategy allowed us to assess the loss of menin in liver, a tissue not normally predisposed to developing tumors in humans or mice with heterozygous *MEN1* loss of function mutations. Progeny that were homozygous for a floxed *Men1* allele and expressing *Cre* showed a nearly complete loss of *Men1* mRNA and corresponding protein levels in the liver, yet the overwhelming majority of menin-null livers were histologically normal and remained tumor free. These findings suggest that in liver, menin's tumor suppressor function or functions are dispensable.

Menin has been reported to interact with a multitude of proteins including JunD, SMAD family members, Pem, NFκB, FANCD2, RPA2, NMMHC II-A, GFAP, vimentin, and Hsp70 [15]. The diverse functions of the menin partners suggest roles for menin in transcriptional regulation, DNA processing and repair, cytoskeletal organization, and protein degradation, although to date none of the interacting partners have been directly proved important in MEN1 pathophysiology. No convincing evidence of direct binding of menin to DNA has been shown, but menin has been shown to associate with a multimember protein complex whose composition is highly similar to that of the SET1 histone methyltransferase complex of yeast and humans [16,17]. Members of this complex include HCF-2 (host cell factor 2), Rbbp5 (retinoblastoma binding protein 5), WDR5 (WD repeat domain 5), and trithorax proteins MLL1/MLL2 (mixed-lineage leukemia) and ASH2 (absent, small, or homeotic). The menin-HMT–associated complexes promote methylation of histone H3 tails at lysine 4 (H3 K4), an epigenetic mark that is generally associated with active gene transcription. At the time of this study, five target genes positively regulated by menin and MLL have been reported in various tissues [16–19]. These targets include the clustered homeobox genes, *HOXA9, c6,* and *c8,* and two cyclin-dependent kinase *(CDK)* inhibitors involved in cell cycle regulation, *p27^Kip1^* and *p18^Ink4c^.*

It has been hypothesized that menin mediates its tumor suppressor action by regulating histone methylation in promoters of *HOX* genes and/or *p18, p27,* and possibly other *CDK* inhibitors [18,19]. Consistent with this hypothesis, H3 K4 methylation and expression of *p18* and *p27* were shown to be dependent on menin in pancreatic islets [18]. An additional clue for a role for *p18* and *p27* in MEN1 pathophysiology comes from studies in knockout mice [20]. The simultaneous loss of *p18* and *p27* in mice leads to a tumor spectrum that is similar to that in human MEN1 and MEN2 patients, including tumors in the pituitary, parathyroid, thyroid, endocrine pancreas, stomach, and duodenum. These findings raise critical questions regarding the function of menin and the basis of endocrine-tumor formation in MEN1. (1) Does menin mediate its tumor suppressor action by governing the expression of *HOX* and cell cycle genes alone or through additional key targets that have not yet been identified? (2) Does menin only target genes whose transcription is trithorax dependent, or can menin also target genes independently of MLL and its associated HMT complex members? (3) Could the specific bias for endocrine tumor formation in MEN1 result from differences in distinct genes that are targeted by menin in the endocrine tissues?

To gain a more complete understanding of the action of menin tumor suppression, we identified the genomic occupancy of menin and two associated HMT complex members, MLL1 and Rbbp5, using chromatin immunoprecipitation coupled with DNA microarray analyses (ChIP-chip) [21]. Given the evidence that the menin-HMT complex methylates histone H3 at lysine 4 [16,18], we also mapped the positions of H3 K4 (trimethylation) by ChIP-chip. Our data in HeLa S3, HepG2, and human pancreatic islet cells indicate that menin occupies the promoter regions of thousands of human genes, suggesting that menin acts as a global regulator of transcription. Menin occupancy frequently coincides with MLL1, Rbbp5, and H3 K4, but menin can also target promoters independently of the HMT complex. Factor occupancy generally correlates with high gene expression, and loss of menin in pancreatic islets, by and large, does not affect transcript levels. Last, we present a model for tumor suppression by MEN1 and offer insights into the endocrine tumor bias observed in MEN1 patients.

## Results

### Pattern of Menin Binding at *HOX* Clusters and Elsewhere

**Figure 1:**
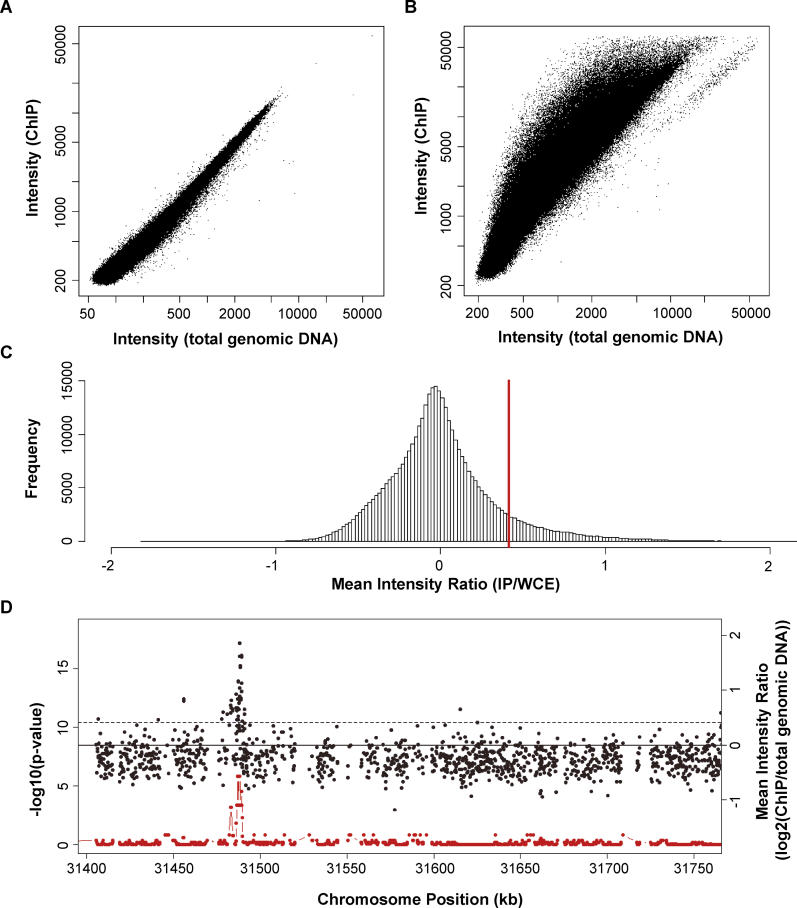
Strategy Used to Analyze Data from Tiled Microarrays (A and B) Scatterplots of single-array intensities of oligonucleotides obtained from chromatin immunoprecipitation with menin antibodies (B) and a control with no antibody (A). Successful experiments are identified as those that show enrichment of multiple probes in the Cy5 channel (chromatin immoprecipitated DNA) over the Cy3 channel (total genomic DNA). (C) Histogram of mean intensity ratios (log2 scale) from menin chromatin immunoprecipitations from three biological replicates. The distinct tail at the right-hand end corresponds to DNA fragments enriched by menin-ChIP. Genomic sites enriched for factor binding are identified using a computer program called ACME. ACME first sorts probes by their genomic location, and then slides a window of user-defined size along tiled regions. ACME then tests whether each window contains a higher than expected number of oligonucletide probes above a user-defined threshold, and then assigns a *p*-value to each probe on the array. For all arrays used in this study, the window was set to 1,000 bp and the threshold at 90% (indicated by the red bar). (D) Plots showing data before and after processing by ACME. Points in black represent the mean intensity ratio of oligonucleotide probes (right *y* axis). Points in red indicate corresponding significance values for each data point reported by ACME (left *y* axis). True-positive signals are represented by multiple probes that cluster within a given window. Single probes that yield high intensity ratios most likely represent noise and are automatically filtered out by the windowing/threshold analysis.

We set out to investigate the genomic occupancy of menin using ChIP-chip. Recent evidence suggesting that menin associates with an SET1-like HMT complex to regulate *HOX* gene expression [16,17] prompted us to design a microarray containing oligonucleotides tiled at high density across the *HOX A, B, C,* and *D* loci. To determine if menin preferentially binds to genes that contain homeobox domains, our arrays also included oligonucleotides corresponding to more than 100 homeodomain-containing genes located outside of the four *HOX* clusters. We also tiled probes corresponding to approximately 20 megabases (Mb) of random sequence on Chromosome 7, to assess menin occupancy in regions located at large distances from genes. As a pilot effort, we hybridized these HOX-centered microarrays with menin-chromatin immunoprecipitated DNA from HeLa S3 cells. A computer program incorporating a sliding window and threshold approach, ACME (Algorithm for Capturing Microarray Enrichment), was used to identify genomic sites enriched for menin-binding (Figure 1).

**Figure 2:**
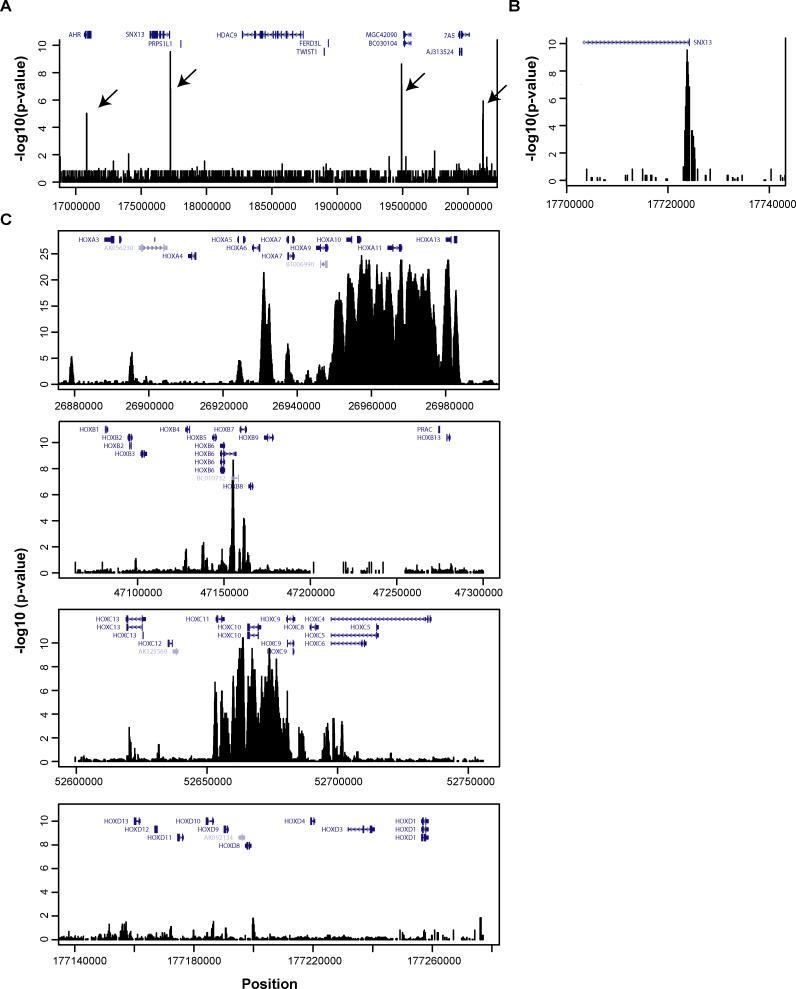
General Pattern of Menin Occupancy in HeLa S3 Cells (A) Tiled oligonucleotides corresponding to a random 3 MB on Chromosome 7. Four positive signals indicative of menin occupancy are denoted by the arrows and are easily distinguishable from background noise. (B) Expanded view of one positive signal from (A) reveals multiple oligos with significant *p*-values clustered solely at the transcriptional start site of *SNX13* (Sorting Nexin 13). (C) Pattern of menin occupancy at each of four homeobox clusters.

Hybridizations to these HOX arrays with menin-chromatin immunoprecipitated DNA from HeLa S3 cells revealed striking differences between menin occupancy at the clustered *HOX* genes as compared to elsewhere. For genes located outside the four *HOX* clusters, menin was frequently localized to 5′ promoter regions, at or very near transcriptional start sites (Figure 2A and 2B). Little to no signal was detected in intragenic regions, or at 3′ untranslated regions. Menin occupancy at the *HOX* clusters was more extensive, with broad footprints that spanned intergenic and intragenic portions of *HOX* genes (Figure 2C). Specifically, within the *HOX A* cluster, menin sites clustered at *HOXA6, A10, A11,* and *A13*. Extensive binding to the *HOX C* cluster was also detected, with broad signals that extended across *HOXC11, C10, C9,* and portions of *C4* and *C6*. Menin sites were also detected near *HOXB6* and *HOXB7,* but the pattern of binding was less extensive than that detected at the *HOX A* and *C* loci. No significant menin binding was detected at the *HOX D* locus. Menin sites were also detected at several other homeobox genes located outside the four *HOX* clusters, but similar to other non-HOX loci, binding was localized to 5′ promoter regions (unpublished data). Our finding that menin binds to *HOXA9, C6,* and *C8* is consistent with prior studies in which these three *HOX* genes were identified by standard ChIP-PCR [16,17] and supports the reliability of the ChIP-chip methodology used here for detecting genomic binding events by menin.

### Genome-Wide Distribution of Menin in Multiple Cell Types

**Figure 3:**
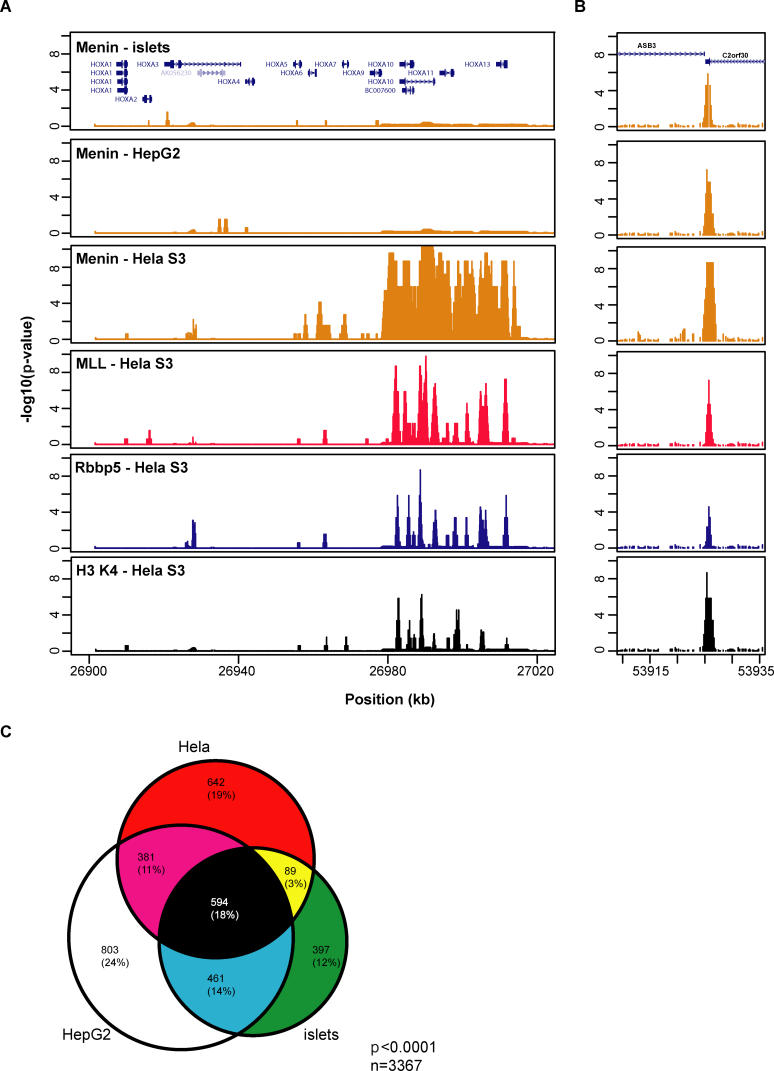
Occupancy of Menin, MLL, Rbbp5 Frequently Overlaps with Trimethylation of Lys4 at Histone H3 (A) Compared to occupancy at the *HOX A* cluster in HeLa cells, factor occupancy in HepG2 cells and pancreatic islets is nearly absent. (B) Overlap of factors at one representative locus, *ASB3* (Ankyrin repeat- and Socs Box-containing protein 3). (C) Venn diagram showing the overlap of menin-bound promoters in HeLa S3, HepG2, and pancreatic islets. Promoters included in the tally had a confidence threshold of *p* < 0.0001.

The results of the pilot study above indicate that, except for the *HOX* clusters, menin occupancy is highly specific for 5′ regions of genes. Based on these findings, we rationalized that menin sites could be identified on a near genome-wide scale by interrogating only those regions of DNA that correspond to the 5′ portions of genes. We therefore designed a second DNA microarray containing tiling oligonucleotides across the 5′ regions of approximately 20,000 human genes. Given the binding pattern of menin at the *HOX* clusters, this array also contained tiled oligos across all four clustered *HOX* loci, as well as the complete sequence of 381 genes (−5,000 bp relative to the transcription start site to +5,000 bp relative to the transcription stop site) that showed evidence of menin occupancy on previous arrays (PCS, unpublished data). These promoter arrays were hybridized with menin-chromatin immunoprecipitated DNA from human islets, HeLa S3 and HepG2 cells. To assess if the bias for endocrine-tumor formation in MEN1 might be related to differences in distinct target genes in endocrine cells, we compared sites occupied in HeLa S3 and HepG2 cells to those identified in human pancreatic islet cells. As expected, hybridizations with menin-chromatin immunoprecipitated DNA from HeLa S3 cells revealed broad signals at the *HOX* clusters and promoter-specific signals at most other loci (Figure 3A and 3B). In HepG2 and pancreatic islet cells, the vast majority of menin sites were also detected in promoter regions, at or near transcriptional start sites. Notable differences in the pattern of menin binding were detected at the *HOX* clusters. Whereas multiple menin sites were detected at the *HOX A* and *C* clusters in HeLa cells, relatively little signal was detected at these clusters in HepG2 cells and pancreatic islets (Figure 3A and unpublished data). By using a stringent selection criterion of *p* < 0.0001, we found that menin occupies 1,706 sites in HeLa S3 cells, 2,239 sites in HepG2 cells, and 1,541 sites in pancreatic islet cells. Sites that were concordant and discordant between each cell type are depicted in a Venn diagram (Figure 3C). The finding that menin binds to a broad range of human promoters in all three cell types suggests that menin has a global role in transcription in both endocrine and non–endocrine-derived cells.

### Overlap of Genomic Occupancy of Menin, MLL, Rbbp5, and the H3 K4 Trimethyl Mark

Based on published evidence that menin associates with a HMT complex containing MLL, Ash2L, WDR5, and Rbbp5, we set out to determine if the genomic occupancy of menin coincides with two other members of this complex, MLL1 and Rbbp5. Given the ability of the SET1/MLL complex to catalyze the trimethlyation of histone H3 at lysine 4, we also mapped the genomic distribution of the H3 K4 trimethyl mark. Results for the *HOX A* cluster are shown in Figure 3A, where the binding patterns for menin, MLL1, Rbbp5, and H3 K4 are seen to be similar, although menin shows a more contiguous binding pattern across *HOXA9, A10, A11,* and *A13*. For non-*HOX* promoters, different binding combinations were seen for different promoters; for illustration, Figure 3B displays a promoter where all factors bind near the transcriptional start site, but that was not always the case.

**Figure 4:**
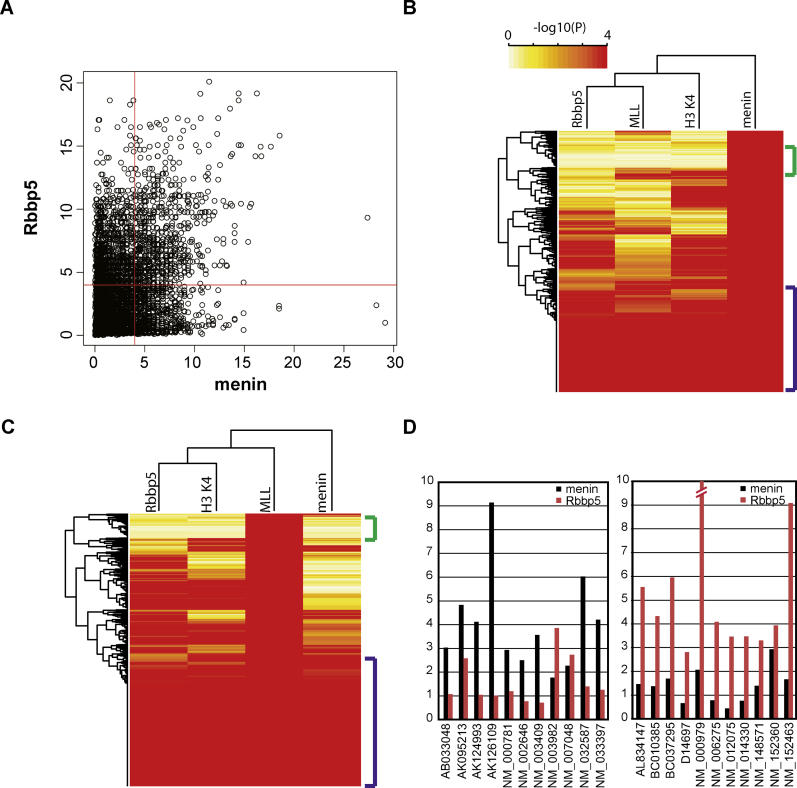
Menin Sites Can Be Bound in the Presence or Absence of HMT Complex Members (A) Comparison of promoters bound by menin and Rbbp5 showing the broad range in signal intensity for each factor. The −log10 *p*-value for each site is plotted on the *x* and *y* axes. Vertical and horizontal lines represent confidence thresholds at *p* < 0.0001. Points in the lower left quadrant represent promoters that are bound by neither menin nor Rbbp5. Points in the upper right quadrant represent promoters occupied by both menin and Rbbp5. Plots comparing menin to MLL and H3 K4 appeared similar (unpublished data). (B and C) Heat maps illustrating the overlap of factor-occupied promoters. Promoters bound by either menin (B) or MLL (C) at the *p* < 0.0001 confidence threshold in HeLa S3 cells were plotted with corresponding *p*-values for each indicated factor. The heat maps reveal subsets of genes that are bound by all factors (blue brackets), in addition to subsets that are bound by menin or MLL but not the other factors (green brackets). (D) Real-time PCR validation of randomly selected promoters determined by ChIP-chip to be bound by menin and not Rbbp5 (left), and vice versa (right). Each pair of bars corresponds to a unique promoter region.

It is not uncommon for ChIP-chip data to reveal dramatic differences in the intensity of hybridization signals at multiple loci [22]. While some loci appear highly enriched, others appear modestly enriched, and other loci entirely lack signal. Differences in signal intensities could reflect true differences in occupancy of the factor or could represent slight differences in amplification or hybridization of chromatin immunoprecipitated DNA. Because of the broad dynamic range in signal, assessing the overlap between different factors at multiple loci at a given significance threshold locus can be challenging (Figure 4A). To depict the overlap more accurately between sites occupied by each factor, we selected promoters that were occupied by menin and MLL1 at high confidence (*p* < 0.0001), and then plotted the corresponding *p*-values for each associated factor as a heatmap. Heatmaps from all three cell types indicate that a large proportion of promoters are bound by all factors (Figure 4B and 4C). The data also reveal distinct sets of promoters occupied by menin and not MLL1 or Rbbp5, and vice versa. Setting stringent cutoffs for bound (*p* < 0.0001) and unbound (*p* > 0.1), the percentage of menin sites that were also bound by MLL1 and Rbbp5 were as follows: 49.3% in HeLa S3 cells; 56.7% in HepG2 cells, and 46.1% in pancreatic islets. The percentage of menin sites that did not coincide with both MLL1 and Rbbp5 was lower: 21.7% in HeLa cells, 9.5% in HepG2 cells, and 2.4% in pancreatic islets. These results suggest that menin targets promoters in cooperation with HMT complex members MLL1 and Rbbp5, but that menin can also occupy promoters in cooperation with other unidentified factors that may not be part of the HMT complex.

### Validation of Factor Binding by Real-Time PCR

**Figure 5:**
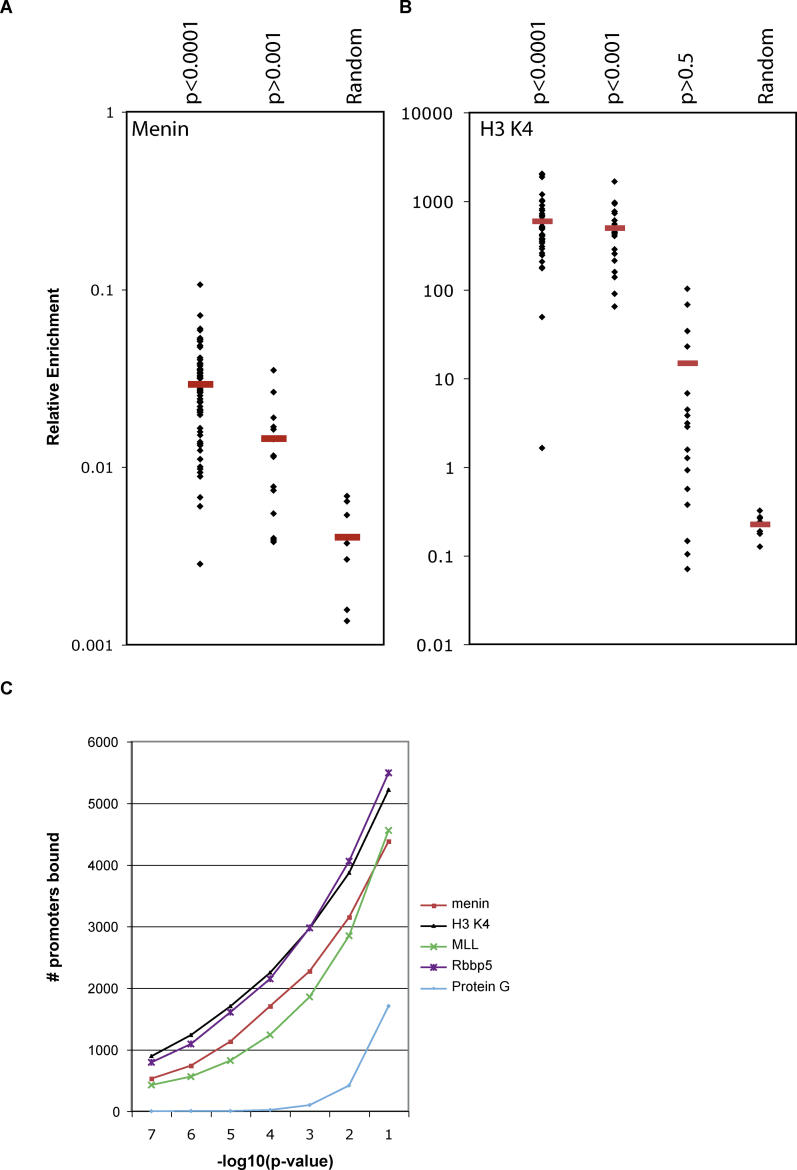
Enrichment of Selected Genomic Regions Real-time PCR testing of genomic regions from menin (A) and H3 K4 (B) chromatin immunoprecipitations. Median values are indicated by the red bars. Enrichment values for menin are less than 1 because C_t_ values for the menin ChIP-PCR were higher than that for total genomic DNA (see Materials and Methods). (C) Number of promoters bound by each factor in HeLa S3 cells at various *p*-values. Compared to a negative control experiment in which no antibodies were used in the ChIP (protein G), significant enrichment of promoters was detected for each factor analyzed.

We used a standard approach that combines conventional ChIP and real-time PCR to assess the reliability of the ChIP-chip method and our approach for analysis of the raw microarray data (Figures 4D and 5). We arbitrarily selected 67 promoter regions that were identified by our windowing and threshold analyses to be enriched for menin binding at *p* < 0.0001. These sites were compared to 15 random promoter regions (0.001 < *p* < 1) and seven random regions of the genome located outside of promoter regions. On average, promoters that had a *p-*value of <0.0001 were enriched 7.25-fold over random regions (Figure 5A). Furthermore, of the 67 sites that were shown to be enriched by ChIP-chip (*p* < 0.0001), 64 (95.5%) were confirmed by real-time PCR to have enrichment ratios that were greater than those detected for the randomly selected amplified regions. These data indicate that sites we determined to be enriched at *p* < 0.0001 by ChIP-chip reliably detect enriched sites of menin binding. Many factor-bound sites probably also exist within the *p* = 0.01 to 0.001 confidence interval, but negative control studies suggest that false-positive rates increase as *p*-values rise above 0.0001 (Figure 5C). Relative enrichment using antibodies to H3 K4 was also tested (Figure 5B). Consistent with the tight physical association between DNA and histones, enrichment of H3 K4–bound sites was greater than that for menin sites.

### Comparison of ChIP-Chip and Gene Expression

**Figure 6:**
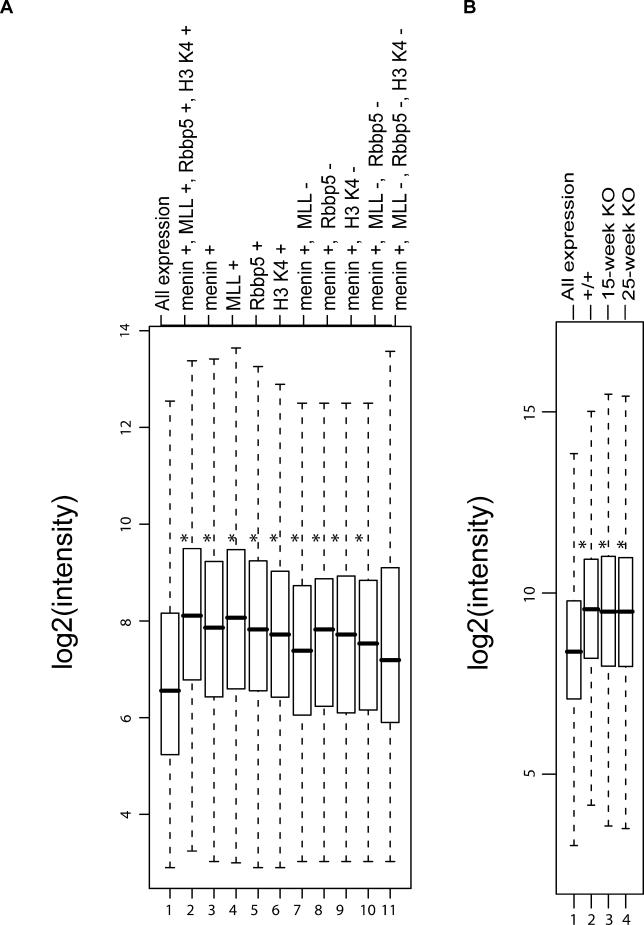
Factor Occupancy Correlates with High Gene Expression but Absence of Menin Does Not Generally Affect Gene Expression Levels (A) Overall expression of genes for which we had both expression and binding data in HeLa S3 cells was plotted (lane 1, “All expression”) and compared to the expression of genes whose promoter regions were bound (*p* < 0.0001) by each of the factors indicated at the top. Asterisks denote significance (*p* < 0.0001) as determined by two-tailed *t*-test analyses between each set of factor-bound genes compared to all genes. Box plots comparing ChIP-chip and expression data from HepG2 and pancreatic islets revealed similar correlations between expression and factor occupancy (unpublished data). (B) Overall expression of genes in islets isolated from wild-type control mice (lane 1, “All Expression”) compared to those normally bound by menin in wild-type islets (lane 2) and those normally bound by menin in islets from 15-wk (lane 3) and 25-wk (lane 4) mice that are conditionally null for *Men1* (genotype: *RIP-cre; Men1* ^−*/*−^).

The ChIP-chip data in all three cell types indicate that menin occupancy frequently coincides with trimethylation of histone H3 at lysine 4, a mark that is generally associated with a transcriptionally active state of chromatin. These results prompted us to assess whether factor occupancy correlates with high levels of gene expression. A systematic comparison between ChIP-chip and expression data in all three cell types revealed that genes whose promoters are bound by menin correlate with higher mRNA expression levels (Figure 6A and unpublished data). This was true for gene subsets both where menin bound concordantly with MLL and Rbbp5 and where menin bound without these factors. These data suggest that menin, in both the presence and absence of the HMT complex, is generally associated with transcriptionally active promoters.

### Comparison of ChIP-Chip and Expression Data from Mice That Are Conditionally Null for *Men1*

The ChIP-chip and expression data indicate that menin targets a broad range of promoters that are generally transcriptionally active. Given these findings, one might predict that menin may function to maintain steady-state gene expression and that loss of menin might lead to reduced expression of genes normally targeted by menin and the HMT complex. To test whether menin-targeted genes show alterations in expression in cells that lack menin, we determined in vivo expression changes incurred upon menin loss in a mouse model of MEN1. Specifically, we compared human ChIP-chip data to expression data from islets isolated from wild-type mice or from mice that were conditionally null for *Men1* in the beta cells (genotype: *RIP-cre; Men1^loxP/loxP^*). Islets from these mice develop tumors long after (30 to 50 wk) homozygous inactivation of the *Men1* alleles and thus provide the opportunity to detect expression changes more closely related to menin loss than at later stages of neoplastic transformation, when secondary activating or inactivating mutations have accumulated. Microarray analyses were performed using RNA from highly purified pancreatic islets isolated from 15- and 25-wk mice that were conditionally null for *Men1.* Controls included islets from age- and sex-matched wild-type and *RIP-cre* transgenic animals. At 15 wk, *Men1* ^−*/*−^ islets appear relatively normal in size and morphology. By 25 wk, the majority of *Men1* ^−*/*−^ pancreatic islets appear hyperplastic and occasionally contain small adenomatous lesions [12].

Similar to the results of previous comparisons between ChIP-chip and gene expression data, genes whose promoters were bound by menin (*p* < 0.0001) correlated with high expression in wild-type mouse islets (Figure 6B). Contrary to what one might predict given the broad range of promoters targeted by menin, we did not detect significant differences in the expression of menin-targeted genes in 15- and 25-wk menin-null islets compared to wild-type (Figure 6B). Since overall expression levels remained generally unchanged in the null state, we turned our attention to the exceptions. One hundred eighty-four individual genes whose promoters were bound by menin in wild-type human islets showed alterations in expression in *Men1* null mouse islets at the 15-wk time point (Figure S1). Of these 184 genes, 110 were upregulated in the absence of menin and 74 were downregulated. Consistent with the prior evidence suggesting that menin positively regulates *p18* in islets [18], *p18 (CDKN2C)* was found to be significantly reduced in expression in *Men1* ^−*/*−^ islets.

### Analysis of *Hlxb9*

Given the specific bias for endocrine tumor formation in MEN1, we focused on genes that were bound by menin in islets and not in HeLa S3 or HepG2 cells, and whose expression was altered in menin-null islets. Twenty-four such genes were found; 12 of these were upregulated in menin-null islets compared to wild-type islets and 12 were downregulated. Of these 24 genes, only seven were significantly different at both the 15- and 25-wk time points. One of these candidates was the transcription factor *Hlxb9*. *HLXB9* encodes HB9, a homeodomain protein predominantly expressed in adult pancreas and developing motor neurons [23,24]. Of particular interest, *HLXB9* is key to normal pancreas development and function, and mice that are homozygous null for *Hlxb9* fail to develop the dorsal lobe of the pancreas [25,26]. The remnant *Hlxb9* ^−*/*−*-*^ pancreas has small islet cells, with reduced numbers of insulin-producing beta cells. Early overexpression of *Hlxb9* in pancreatic epithelium results in a complete disruption of pancreatic development [27]. In humans, dominantly inherited loss of function mutations in *HLXB9* causes Currarino syndrome, a developmental disorder characterized by partial sacral agenesis, a presacral mass, and anorectal and urogenital malformations [28–30]. Activation of *HLXB9* expression may be induced by chromosomal rearrangements in acute lymphoblastic leukemia [31,32].

**Figure 7:**
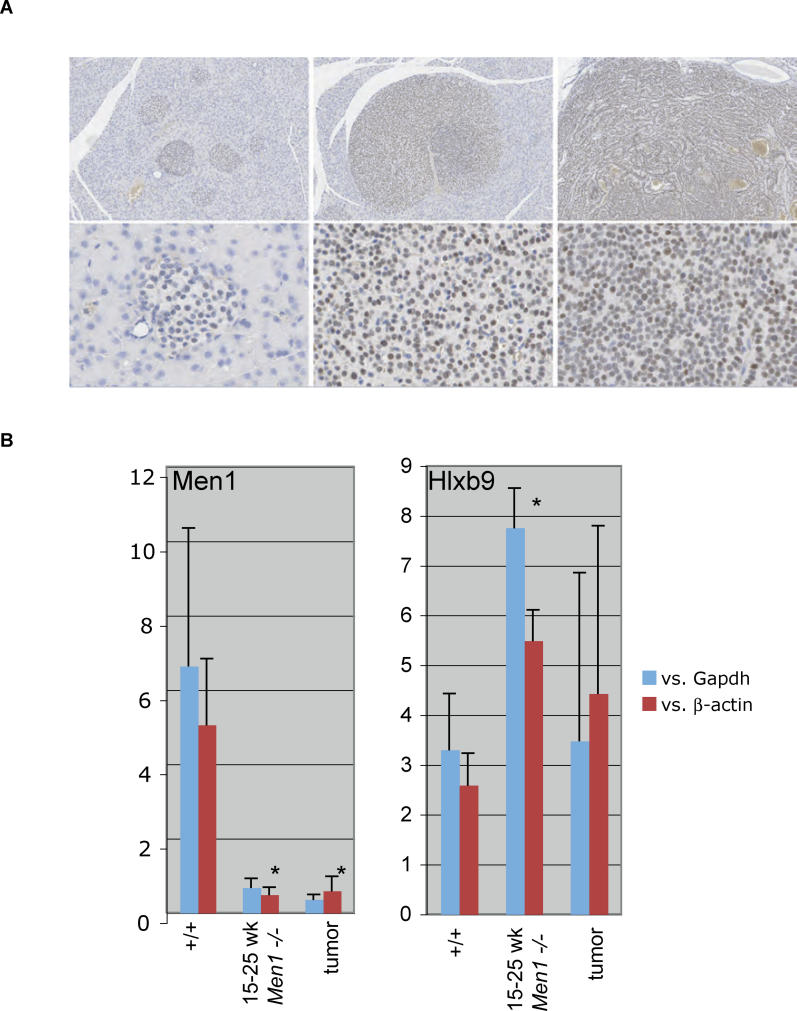
Hb9 Expression Is Elevated in Islets from Conventional and Conditional *Men1* Knockout Mice (A) Pancreatic sections from an 18-mo conventional *Men1* knockout mouse *(Men1^+/^* ^−^ *)* were analyzed for Hb9 expression by immunohistochemistry. Brown stain corresponds to positive Hb9 signal. Compared to normal-size islets (left), an atypical hyperplastic islet (middle) and tumor (right) show increased Hb9 expression. All pictures were taken from different regions of the same section, all at the same exposure. Top panels: ×100 magnification, bottom panels: ×200 to ×400. (B) Real-time PCR analysis of *Men1* and *Hlxb9* expression in *Men1^+/+^* and *Men1* ^−*/*−^ islets from conditional animals. Blue bars indicate expression relative to *Gapdh;* red bars, expression relative to beta-actin; +/+, average expression of five control mice (three *RIP-Cre; Men1^+/+^*, two *Men1^+/+^*); 15–25 wk *Men1* ^−*/*−^, average of one pool of conditional islets at 15 wk (five) and three individual preps from 25-wk *Men1* ^−*/*−^ islets *(RIP-cre; Men1^loxP/loxP^);* and tumor, average expression of five tumors from conditional *Men1* knockout mice *(RIP-cre; Men1^loxP/loxP^)*. Asterisks denote statistical significance (*p* < 0.02) for both blue and red bars as determined by two-tailed *t*-tests.

To test the possibility that menin regulates beta cell–specific expression of *Hlxb9* and to validate the microarray results described above, we measured gene expression by RT-PCR in *Men1* ^−*/*−^ pancreatic islets and islet tumors isolated from a cohort of conditionally null *Men1* mice (Figure 7). Compared to islets isolated from wild-type mice, expression of *Hlxb9* was significantly increased in 15- and 25-wk *Men1* ^−*/*−^ islets. Consistent with these findings, immunohistochemical analyses in a conventional *Men1* knockout animal revealed increased Hb9 expression in atypical hyperplastic islets and tumor tissue. Collectively, these findings suggest activation of *Hlxb9* in response to menin loss might contribute to islet beta cell tumorigenesis.

### Menin Occupancy at *p18* and *p27* Loci

Recent reports suggesting that menin can positively regulate *p18* and *p27* [18] prompted us to investigate ChIP-chip and expression data at these two loci. We detected intense ChIP hybridization signals for menin, MLL, and H3 K4 trimethylation at the promoters of both *p18* and *p27* in HeLa S3, HepG2, and pancreatic islet cells. Menin, MLL, and H3 K4 occupancy at *p18* and *p27* loci also coincided with Rbbp5 binding in HeLa and HepG2 cells but not in pancreatic islets. Consistent with previous findings [18], we detected reduced expression of *p18* in *Men1* ^−*/*−^ islets by microarray analyses (Figure S1), and this was validated by real-time RT-PCR (unpublished data). Alterations in *p27* expression levels were not detected in null islets.

Given that menin was found by ChIP-chip to occupy *p18* and *p27* in all three cell types, we next set out to determine if the absence of menin affects *p18* and *p27* expression levels in a nonendocrine tissue. In an effort to gain insights into tissue specificity in MEN1, we previously used the *Cre-lox* system to generate mice that are homozygous null for *Men1* in liver [14]. Mice with *Men1* ^−*/*−^ livers showed a nearly complete loss of *Men1* mRNA corresponding protein levels, yet the menin-null livers remain tumor free. To assess if menin maintains *p18* and *p27* expression in liver, we measured *p18* and *p27* gene expression by RT-PCR in liver tissues isolated from these mice. Compared to wild-type control livers, the expression of *p18* and *p27* in *Men1*-null livers was reduced 89% and 63%, respectively (unpublished data). Collectively, these data support the conclusion that menin enhances expression of *p18* and *p27* in both pancreatic islets and liver. Since the absence of menin gives rise to tumors only in endocrine pancreas, not in liver, mechanisms beyond simple dysregulation of *p18* and *p27* must be involved.

## Discussion

We set out to investigate menin's role as a transcriptional regulator by identifying genes bound by and dependent on menin. Prior to this study, the only genes reported to be targeted by menin and MLL were *Hoxc6, Hoxc8, HOXA9, p27,* and *p18*. Our findings indicate that menin does not only target specific classes of genes like *HOX* and *CDK* inhibitors but that menin also targets a very broad range of promoters in multiple tissues. This expands the realm of menin-targeted genes several hundred-fold. We find that menin occupancy frequently coincides with members of the HMT complex, MLL1 and Rbbp5, as well as the H3 K4 trimethyl mark, raising the possibility that menin functions as a general transcriptional regulator that helps maintain stable gene expression. Averaged over thousands of genes, menin correlates with higher gene expression, but overall expression levels do not decrease upon loss of menin in null islets. Surprisingly, menin binds many regions of the genome independently of the HMT complex members, suggesting that menin may regulate transcription by cooperating with other, currently unknown proteins. Possible candidates include JunD, SMADs, NFκB, or other menin-associated transcription factors not part of the SET1/MLL complex.

A fundamental question for many tumor suppressor syndromes is the tissue specificity of tumor formation. Menin is expressed in nearly all tissues. Why do MEN1-associated tumors arise primarily in endocrine organs? Could the interactions between menin, MLL, and *HOX* gene expression suggest an explanation for the endocrine specificity?

MLL is an important regulator of *HOX* gene expression, and leukemic chromosomal translocations involving *MLL* appear to act by creating chimeric fusion proteins that constitutively activate *HOX* genes [33–35]. The persistent expression of *HOX* genes results in a failure of terminal differentiation, and studies in mice have shown that overexpression of certain *HOX* genes, *Hoxa9* and *Meis1* in particular, leads to leukemogenesis [36,37]. Previous studies have also shown that menin can positively regulate *HOXA9* in HeLa cells and *Hoxc6* and *Hoxc8* in developing mouse embryo [16,17]. Collectively, these findings have led to the enticing hypothesis that tumorigenesis in MEN1, like leukemogenesis, is related to dysregulation of *HOX* genes normally bound by menin and MLL. If this hypothesis is valid, however, we would have expected to see menin occupancy at the *HOX* clusters in pancreatic islets. While we did detect extensive binding of menin (and MLL) to the *HOX A* and *C* clusters in HeLa S3 cells, virtually no signal was detected in either HepG2 cells or pancreatic islets. Moreover, the clustered homeobox genes are expressed at almost undetectable levels in pancreatic islets, and none of them significantly change in expression following loss of menin. Given these findings and the contrasts of menin function in myeloid versus endocrine tumors, we believe it is unlikely that dysregulation of *HOX* genes in islet cells contributes to MEN1 islet neoplasms.

If *HOX* genes are not the mediator, then how does menin loss lead to endocrine tumors? Menin was recently proposed to regulate pancreatic islet growth by promoting histone methylation and expression of cyclin-dependent kinase inhibitors *p27* and *p18* [18,19]. Moreover, the simultaneous loss of *p18* and *p27* in mice was shown to lead to development of tumors in many of the same endocrine tissues as humans with inherited mutations in the *MEN1* and *MEN2* genes [20]. These studies argue for a major role for *p18* and *p27* in the tissue-specific tumor phenotype in MEN1. Specifically, loss of menin prevents H3 K4 methylation at the promoters of *p18* and *p27,* reducing their expression, and ultimately abrogating their inhibitory effect on cyclin-dependent kinases (CDK2 and CDK4) and resulting in unrestrained cell growth. Consistent with this hypothesis, we detected menin at the promoters of *p18* and *p27* and reduced *p18* expression in islet cells that were conditionally null for *Men1*. However, *p18* and *p27* concentrations were reduced in *Men1* ^−*/*−^ liver, which is not susceptible to developing tumors in heterozygous *MEN1* patients, or in mice rendered null for menin in liver by a conditional knockout. A separate study showed that menin can regulate *p18* and *p27* expression in fibroblasts [19], yet the phenotype of MEN1 does not include fibrosarcoma. These results indicate that while dysregulation of *p18* and *p27* may contribute to neoplastic transformation in MEN1, these genes alone cannot account for the specific bias for endocrine tumor formation in MEN1.

To identify other possible genetic causes for the tissue specificity of tumor formation, we identified a relatively small number of genes that were both bound by menin and altered in expression in menin-null islets. One of these genes was the developmentally programmed *HLXB9,* which codes for transcription factor HB9. Hb9 expression was significantly elevated in pancreatic islets in the absence of menin, suggesting that menin is a transcriptional repressor of *HLXB9*. It is also noteworthy that *HLXB9* was bound by menin only in islets, and not in HeLa or HepG2 cells. These findings raise the possibility that the specific bias for endocrine tumor formation in MEN1 could result from changes in expression in distinct genes, including *HLXB9,* that are specifically targeted by menin in endocrine tissues.

**Figure 8:**
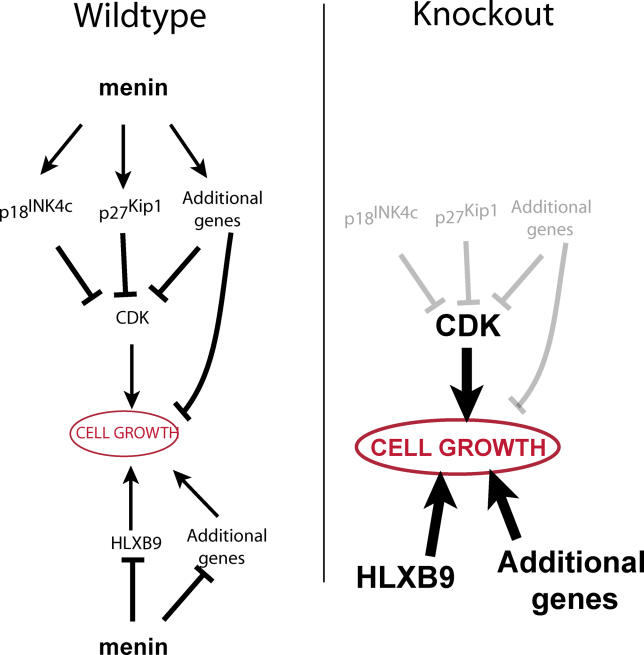
Hypothetical Model of Menin Function See text.

Based on our findings and the work of others, we propose the following model for tumor suppression by menin in pancreatic islets (Figure 8). In both endocrine and nonendocrine tissues, menin mediates transcriptional activation of genes that inhibit cell growth, including, but not limited to, the cyclin-dependent kinase inhibitors *p18* and *p27*. In addition, menin can function as a corepressor of tissue-specific genes that promote cell growth, including *HLXB9*. Menin can mediate its regulatory functions in the presence of the HMT complexes or other menin partners. Endocrine neoplasia as a consequence of menin loss results from the combined effects of decreased *p18* and *p27* and increased *HLXB9* expression. Additional tissue-specific factors that are dysregulated upon menin loss probably also contribute to neoplasia. Similar studies in additional tissues, such as anterior pituitary or parathyroid, may help reveal the complete list of critical targets.

## Materials and Methods

### Antibodies for chromatin immunoprecipitation.

Affinity-purified rabbit polyclonal anti-menin (BL342), anti-Rbbp5 (BL766), and anti-MLL1 (BL1289) antibodies were obtained from Bethyl Laboratories (Montgomery, Texas, United States). The menin antibody recognizes an epitope at the C terminus of menin. The Rbbp5 antibody was raised against a portion of human Rbbp5 encoded within exons 13 and 14. The epitope recognized by anti-MLL1 maps to a region between residues 720 and 780 of MLL1. This epitope is found in the N-terminal 300-kDa fragment generated by proteolytic cleavage. Histone H3 K4 antibody (ab8580; Abcam, Cambridge, United Kingdom) reacts to trimethylated H3 K4 and shows weak reactivity to dimethylated H3 K4.

### Microarrays used for ChIP-chip.

Two different DNA tiling microarray designs were used in this study. Both microarrays were printed by NimbleGen (Madison, Wisconsin, United States). The first array contained 190,181 oligonucleotides (70 nucleotides in length) representing *HOX* clusters *A, B, C,* and *D,* and approximately 230 loci tiled at an average resolution of one oligo every 150 to 350 bp. Roughly half of these 230 loci harbored genes whose protein products contain homeodomains. The remaining half were identified as positive “hits” in a pilot study using a previously described conventional microarray that contained portions of promoter regions of 13,000 human genes (unpublished data). For each of these 230 genes, oligonucleotides spanning −10 kb relative to the transcriptional start site to +10 kb relative to the end of the transcript were tiled. The coordinates for DNA corresponding to the *HOX* clusters based on the July 2003 human genome assembly are as follows: *HOX A:* Chromosome 7: 16,875,358 to 36,981,974; *HOX B:* Chromosome 17: 47,064,003 to 47,300,285; *HOX C:* Chromosome 12: 52,599,536 to 52,755,746; *HOX D:* Chromosome 2: 177,140,087 to 177,277,119. The second tiling microarray we designed contained oligonucleotides tiled across 19,928 promoter regions. The oligonucleotides were distributed at a resolution of approximately one 50 mer every 100 bp (1.5 kb approximately each promoter region). This array also contained tiling oligonucleotides across 381 genes (−5 kb relative to each transcriptional start site to +5 kb relative to the end of each transcript) at an average resolution of one oligo every 180 bp. The array also harbored tiling oligonucleotides across all four *HOX* clusters. The coordinates for DNA corresponding to the *HOX* clusters were based on the May 2004 human genome assembly and are as follows: *HOX A:* Chromosome 7: 26,901,743 to 27,173,542; *HOX B:* Chromosome 17: 43,958,347 to 44,167,626; *HOX C*: Chromosome 12: 52,616,715 to 52,738,384; *HOX D*: Chromosome 2: 176,780,780 to 176,883,479.

### ChIP-chip.

The protocol described here was adapted from previously published studies [21,38]. Briefly, for each ChIP-chip experiment, 1 to 2 × 10^8^ cells were crosslinked with 1% formaldehyde for 20 min at room temperature, harvested, and rinsed with 1× PBS. Cell nuclei were isolated, pelleted, and sonicated. DNA fragments were enriched by immunoprecipitation with factor-specific antibodies. After heat-reversal of the crosslinking, the enriched DNA was amplified by ligation-mediated PCR (LM-PCR) and then fluorescently labeled by using Klenow polymerase and Cy5-labeled dUTP (Amersham Biosciences, Piscataway, New Jersey, United States). A sample of DNA that was not enriched by immunoprecipitation was subjected to LM-PCR and labeled with Cy3-dUTP. ChIP-enriched and unenriched (input) labeled samples were cohybridized to microarrays. Microarrays were hybridized 18 to 20 h at 45 °C, washed according to the protocol described by NimbleGen, and scanned using an Agilent Technologies (Palo Alto, California, United States) microarray scanner. As a pilot study, the first custom-designed “HOX” array was hybridized with menin chromatin immunoprecipitated DNA from HeLa S3 cells from a single experiment. For experiments using the second, more promoter-oriented array design, three biological replicates were performed for experiments in HeLa S3 and HepG2 cells. In purified pancreatic islet preps, menin ChIP-chip was performed in biological quadruplicate, and Rbbp5 and H3 K4 experiments in duplicate. Due to limited availability of primary islet tissue, ChIP-chip in islet cells using antibodies to MLL1 was done once. Islet preparations were treated with formaldehyde in culture media between 1 h and 5 d after isolation from pancreata, which were derived from brain-dead organ donors (National Institutes of Health, Institutional Review Board exemption issued by National Institutes of Health Office of Human Subjects [IRB Exemption No. 3072]). For each ChIP-chip experiment, approximately 30,000 viable islet equivalents were fixed and handled as described above. Islet purity ranged from 40% to 90% with 60% to 90% viability.

### Analysis of ChIP tiling array data.

Raw array data were normalized using bi-weight mean using the NimbleScan Version 2.1 software (NimbleGen Systems). Log2 ratios (cy5/cy3) from biological replicates were averaged. To identify potential sites of enrichment, a window and threshold strategy was used (Figure 1). Briefly, a 1,000-bp window was moved stepwise along the tiled region, centering at every probe. Hybridization signals of probes within each window were tested by χ^2^ analysis to determine if the window contained a higher than expected number of probes. These calculations resulted in a single *p*-value associated with each averaged data point. Analyses performed at different window sizes and thresholds yielded similar results (unpublished data). All raw and processed ChIP-chip data is publicly available from Gene Expression Omnibus (GEO) (http://www.ncbi.nlm.nih.gov/geo). The *p*-value scores for each promoter tested are included. The windowing and threshold program we developed, which we named ACME (Algorithm for Capturing Microarray Enrichment), is available from the authors upon request.

### Real-time PCR validation.

Standard ChIP with menin and trimethyl H3 K4 antibodies was performed on HeLa S3 cells in duplicate. PCR primer pairs were designed to amplify 150- to 200-bp fragments from selected genomic regions. For menin, we compared 67 promoters determined by ChIP-chip to be enriched for menin binding at *p* < 0.0001 to 15 random promoter regions (0.001 < *p* < 1) and seven random regions located outside of promoter regions. For H3 K4, we tested 38 promoters that were determined by ChIP-chip to be highly enriched (*p* < 0.0001), 20 marginally enriched genes (0.0001 < *p* < 0.001), 17 unenriched promoter regions (*p* > 0.5), and seven random regions.

Real-time PCRs were carried out in duplicate on each chromatin immunoprecipitated and input DNA sample using SYBR green PCR mix (Qiagen, Valencia, California, United States) in an Applied Biosystems 7900HT Fast Real Time-PCR machine (Foster City, California, United States). DNA obtained from menin chromatin immunoprecipitations was very limited in quantity and could not be quantitated by standard methods. As a result, unequal quantities of ChIP and input DNA were added to PCRs. To account for the differences in DNA quantity, for every genomic region studied, a ΔC_t_ value was calculated for each sample by subtracting the C_t_ value for chromatin immunoprecipitated sample from the C_t_ value obtained for the input. Raising 2 to the ΔC_t_ power yielded the relative amount of PCR product (relative enrichment). Average values for promoters occupied by either menin or H3 K4 were compared to those calculated for unoccupied promoters and random regions of the genome. All primer sequences are available upon request.

### Comparison of ChIP-chip and expression data.

ChIP-chip data from islet cells were merged to Affymetrix (Santa Clara, California, United States) islet expression data from the Novartis (version 2) data set [39]. HeLa S3 and HepG2 ChIP-chip data were merged to expression data generated in our laboratory using Affymetrix U133A Plus microarrays (HeLa S3 and HepG2). All ChIP-chip and expression datasets were merged by gene symbol. For all three cell types, two replicate datasets were averaged. Average raw intensity values were converted to log base 2 and normalized using the robust multiarray average method (RMA) [40]. Data for genes represented by multiple probes were retained in the dataset. This analysis gave 10,148 genes in islet cells and 10,078 genes in HeLa S3 and HepG2 cells for which we had both binding and expression data.

Microarray analyses were also performed using RNA from highly pure islets prepared from 15-wk-old (*n* = 5) and 25-wk-old (*n* = 3) female mice that were conditionally null for *Men1* (genotype: *RIP-cre, Men1^loxP/loxP^*). Controls included islet RNA prepared from sex-matched wild-type (*n* = 3) and *RIP-cre* (*n* = 2) mice at 15 wk of age. Total RNA isolated from each group of mice was pooled and subjected to two rounds of amplification three times. Biotin-labeled cRNA was then purified, fragmented, and hybridized to MOE430A Affymetrix GeneChip probe arrays containing more than 22,000 probe sets. Two-tailed *t*-tests using Microsoft Excel were used to compare expression values from wild-type and RIP-cre controls to 15-wk conditionally null mice *(RIP-cre, Men1^loxP/loxP^)*. The mouse expression data were merged to the human ChIP-chip data, yielding 5,519 unique genes for which we had both expression and binding data.

### Immunohistochemistry.

The pancreas from an 18-mo heterozygous *Men1* knockout mouse *(Men1^+/^* ^−^ *)* was removed, immediately fixed in 4% paraformaldehyde, and embedded in paraffin. Immunohistochemistry of pancreatic sections from an 18-mo conventional *Men1* knockout mouse *(Men1^+/^* ^−^ *)* was performed following antigen retrieval using previously described antibodies to Hb9 [25].

## Supporting Information

Figure S1 The 184 Genes That Were Found to Separate 15-wk *Men1*-Null Islets from Controls Are Depicted in the Red-Green Color-Coded Plot (*p* < 0.01) Relative expression levels have been pseudo-colored red and green, with red corresponding to high expression and green corresponding to low expression. The color-coded plot to the right of the expression data represents ChIP-chip *p*-value binding data for each factor. Color key: red, *p* < 0.0001; orange, 0.0001 > *p* < 0.001; gold, 0.001 > *p* < 0.01; light yellow, 0.01 > *p* <0.1; white, *p* > 0.1. Twenty-four genes whose promoters were bound by menin exclusively in islets are depicted in red and blue. Those in red show statistically significant differences in expression at both the 15- and 25-wk time points (*p* < 0.01).
(142 KB PDF)

## Abbreviations

ChIP-chip: chromatin-immunoprecipitation coupled with microarray analysis
MEN1: multiple endocrine neoplasia, type I
