# Supplementary material for: Genome-Wide Analysis of Menin Binding Provides Insights into MEN1 Tumorigenesis
Source: PLoS Genet. 2006 Apr 7;2(4):e51. doi: 10.1371/journal.pgen.0020051 (PMC1428788; doi:10.1371/journal.pgen.0020051)
Supplement: Supplementary file 1 [file pgen.0020051.sg001.pdf]

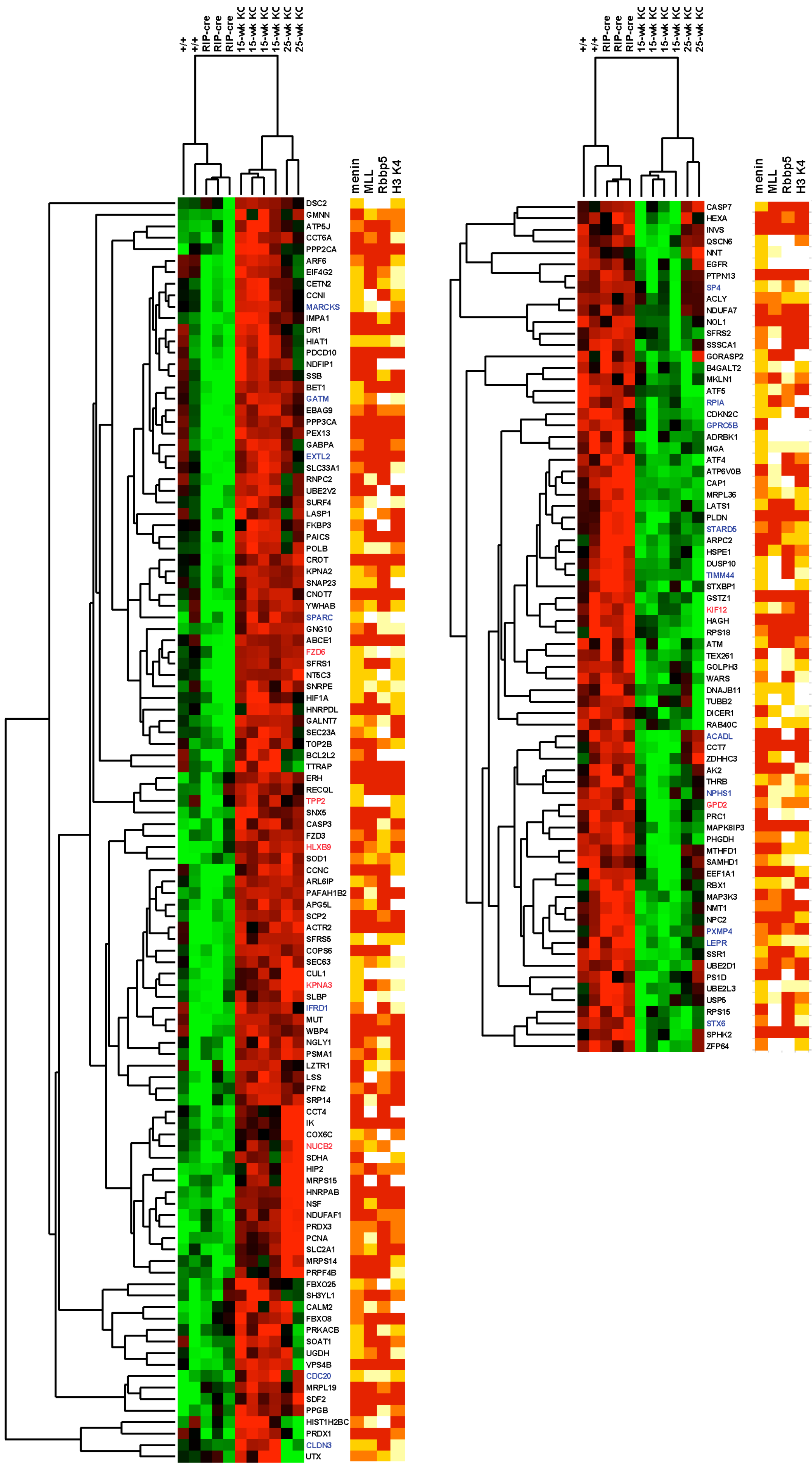
+/+
+/+
RIP-cre
RIP-cre
RIP-cre
15-wk KC
15-wk KC
15-wk KC
15-wk KC
25-wk KC
25-wk KC
menin
MLL
Rbbp5
H3 K4
DSC2
GMNN
ATP5J
CCT6A
PPP2CA
ARF6
EIF4G2
CETN2
CCNI
MARCKS
IMPA1
DR1
HIAT1
PDCD10
NDFIP1
SSB
BET1
GATM
EBAG9
PPP3CA
PEX13
GABPA
EXTL2
SLC33A1
RNPC2
UBE2V2
SURF4
LASP1
FKBP3
PAICS
POLB
CROT
KPNA2
SNAP23
CNOT7
YWHAB
SPARC
GNG10
ABCE1
FZD6
SFRS1
NT5C3
SNRPE
HIF1A
HNRPDL
GALNT7
SEC23A
TOP2B
BCL2L2
TTRAP
ERH
RECQL
TPP2
SNX5
CASP3
FZD3
HLXB9
SOD1
CCNC
ARL6IP
PAFAH1B2
APG5L
SCP2
ACTR2
SFRS5
COPS6
SEC63
CUL1
KPNA3
SLBP
IFRD1
MUT
WBP4
NGLY1
PSMA1
LZTR1
LSS
PFN2
SRP14
CCT4
IK
COX6C
NUCB2
SDHA
HIP2
MRPS15
HNRPAB
NSF
NDUFAF1
PRDX3
PCNA
SLC2A1
MRPS14
PRPF4B
FBXO25
SH3YL1
CALM2
FBXO8
PRKACB
SOAT1
UGDH
VPS4B
CDC20
MRPL19
SDF2
PPGB
HIST1H2BC
PRDX1
CLDN3
UTX
+/+
+/+
RIP-cre
RIP-cre
RIP-cre
15-wk KC
15-wk KC
15-wk KC
15-wk KC
25-wk KC
25-wk KC
menin
MLL
Rbbp5
H3 K4
CASP7
HEXA
INVS
QSCN6
NNT
EGFR
PTPN13
SP4
ACLY
NDUFA7
NOL1
SFRS2
SSSCA1
GORASP2
B4GALT2
MKLN1
ATF5
RPIA
CDKN2C
GPRC5B
ADRBK1
MGA
ATF4
ATP6V0B
CAP1
MRPL36
LATS1
PLDN
STARD5
ARPC2
HSPE1
DUSP10
TIMM44
STXBP1
GSTZ1
KIF12
HAGH
RPS18
ATM
TEX261
GOLPH3
WARS
DNAJB11
TUBB2
DICER1
RAB40C
ACADL
CCT7
ZDHHC3
AK2
THRB
NPHS1
GPD2
PRC1
MAPK8IP3
PHGDH
MTHFD1
SAMHD1
EEF1A1
RBX1
MAP3K3
NMT1
NPC2
PXMP4
LEPR
SSR1
UBE2D1
PS1D
UBE2L3
USP5
RPS15
STX6
SPHK2
ZFP64
